# Isoliquiritigenin Ameliorates Acute Pancreatitis in Mice via Inhibition of Oxidative Stress and Modulation of the Nrf2/HO-1 Pathway

**DOI:** 10.1155/2018/7161592

**Published:** 2018-04-26

**Authors:** Xinnong Liu, Qingtian Zhu, Min Zhang, Tao Yin, Rong Xu, Weiming Xiao, Jian Wu, Bin Deng, Xuefeng Gao, Weijuan Gong, Guotao Lu, Yanbing Ding

**Affiliations:** ^1^Laboratory of Gastroenterology, Affiliated Hospital of Yangzhou University, Yangzhou University, Yangzhou, China; ^2^Department of General Surgery, Affiliated Hospital of Yangzhou University, Yangzhou University, Yangzhou, China; ^3^Department of Gastroenterology, Affiliated Hospital of Yangzhou University, Yangzhou University, Yangzhou, China; ^4^Department of Immunology, School of Medicine, Yangzhou University, Yangzhou, China

## Abstract

Oxidative stress plays a crucial role in the pathogenesis of acute pancreatitis (AP). Isoliquiritigenin (ISL) is a flavonoid monomer with confirmed antioxidant activity. However, the specific effects of ISL on AP have not been determined. In this study, we aimed to investigate the protective effect of ISL on AP using two mouse models. In the caerulein-induced mild acute pancreatitis (MAP) model, dynamic changes in oxidative stress injury of the pancreatic tissue were observed after AP onset. We found that ISL administration reduced serum amylase and lipase levels and alleviated the histopathological manifestations of pancreatic tissue in a dose-dependent manner. Meanwhile, ISL decreased the oxidative stress injury and increased the protein expression of the Nrf2/HO-1 pathway. In addition, after administering a Nrf2 inhibitor (ML385) or HO-1 inhibitor (zinc protoporphyrin) to block the Nrf2/HO-1 pathway, we failed to observe the protective effects of ISL on AP in mice. Furthermore, we found that ISL mitigated the severity of pancreatic tissue injury and pancreatitis-associated lung injury in a severe acute pancreatitis model induced by L-arginine. Taken together, our data for the first time confirmed the protective effects of ISL on AP in mice via inhibition of oxidative stress and modulation of the Nrf2/HO-1 pathway.

## 1. Introduction

Acute pancreatitis (AP) is an acute noninfectious inflammatory disease involving abnormal inflammation of the pancreas. In recent years, the incidence of AP has significantly increased. Most cases of AP are mild and self-limited, but approximately 15–20% of patients develop severe acute pancreatitis (SAP) with up to 30% mortality. Although the clinical treatment of AP has substantially improved, the mortality of patients with SAP is still high and remains a major problem worldwide [1–3].

Oxidative stress is one of the most important pathways of cell injury [4, 5]. Many studies have shown that oxidative stress plays a major role in the pathogenesis of pancreatic tissue injury in a murine model of AP [6, 7]. Clinical and experimental data have shown that oxidative stress is present in the early phase of AP [8]. The injured pancreatic acinar cells and activated immune cells in pancreatic tissue release large amounts of oxygen free radicals, accompanied by increased malondialdehyde (MDA) and decreased superoxide dismutase (SOD) and total glutathione (GSH) levels. Moreover, treatment with antioxidants has been shown to reduce acinar cell necrosis and mitigate the severity of pancreatic tissue injury in various animal models of AP. All the previous findings confirm the critical role of oxidative stress in the pathomechanism of AP [9–11].

Liquorice, a traditional Chinese medicine, has been used for years in clinical practice. Recently, its active ingredients have been found to be effective for many diseases; for example, the compound glycyrrhizin was shown to be an efficacious treatment for hepatic injury in clinical practice [12]. Liquiritigenin (ISL) is a natural flavonoid compound derived from the natural herb licorice root (licorice), which has a chalcone structure (4,20,40-trihydroxy chalcone) [13]. ISL has extensive pharmacological functions, including antitumor, anti-inflammatory, and antiplatelet aggregation activities [14–16]. Furthermore, ISL shows a distinct antioxidative stress effect, protecting animal models of diabetic nephropathy, intracranial hemorrhage, and hepatic from injury [17]. However, the effect of ISL on AP is still unknown. In this study, we aimed to clarify the effect of ISL on AP and investigate the potential underlying mechanisms using two murine models.

## 2. Materials and Methods

### 2.1. Animals

ICR male mice (8 weeks old) weighing between 28 and 32 g were purchased from the Yangzhou University Model Animal Center (Yangzhou, China) and housed in specific pathogen-free (SPF) facilities. Before experimentation, the animals were fed standard rodent chow and water, monitored at a controlled temperature, and maintained under a 12 h light/dark cycle for at least a week. This study was performed with the permission of the Science and Technology Commission of the Affiliated Hospital of Yangzhou University municipality, and all methods were carried out in accordance with the Principles of Laboratory Animal Care (NIH publication number 85Y23, revised 1996).

### 2.2. Reagents

ISL and resveratrol (RES) were purchased from Aladdin (Aladdin Bio-Chem Technology Company, Shanghai, China); caerulein was purchased from AnaSpec (AnaSpec Inc., Fremont, USA); L-arginine and zinc protoporphyrin (ZnPP) were obtained from Sigma (Sigma-Aldrich, St. Louis, MO, USA); a nuclear protein extraction kit was purchased from Nanjing KeyGen (Nanjing KeyGen Biotech Co. Ltd., Nanjing, China); antinuclear factor erythroid 2-related factor 2 (Nrf2) and heme oxygenase (Ho-1) antibodies were purchased from Abcam (Cambridge, UK); anti-Lamin B1 antibody was purchased from Proteintech (Proteintech Group Inc., Chicago, USA); and anti-*β*-actin antibody was purchased from Santa Cruz Biotechnology (Santa Cruz, CA, USA). Goat antirabbit and rabbit antimouse secondary antibodies were purchased from Abcam (Abcam, Cambridge, UK). The Nrf2 inhibitor ML385 was purchased from Medchem Express (MCE Co. Ltd., Shanghai, China); lipase kits were purchased from Nanjing Jiancheng (Nanjing Jiancheng Corp., Nanjing, China), and amylase kits were purchased from Zhongsheng Beikong (Zhongsheng Beikong Bio-Technology, Beijing, China). The products of oxidative stress test kits, including MDA, SOD, and GSH, were purchased from Nanjing KeyGen (Nanjing KeyGen Biotech Co. Ltd., Nanjing, China.). Dihydroethidium (DHE) and 4′,6-Diamidino-2-Phenylindole, Dihydrochloride (DAPI) staining solution were purchased from Servicebio (Wuhan Servicebio Technology Co. Ltd., Wuhan, China.). Interleukin- (IL-) 6 and IL-1*β* kits were purchased from Ebioscience (Affymetrix eBioscience, Santiago, USA).

### 2.3. Preparation of the MAP Model and ISL/RES Intervention

As previously reported, the MAP model was induced by intraperitoneal (i.p.) injection of caerulein (50 *μ*g/kg, interval of one hour, 10 times). For determination of the dynamic changes in the oxidative stress response in AP, mice were sacrificed at 0 h, 6 h, 12 h, 24 h, and 3 d after the first injection of caerulein [18].

ISL/RES treatment: experimental mice were randomly assigned to six groups: the vehicle, caerulein, low-dose ISL (50 mg/kg), middle-dose ISL (100 mg/kg), high-dose ISL (200 mg/kg), and RES (100 mg/kg) groups [19]. All groups except for the vehicle group were injected with caerulein to induce the MAP model. ISL and RES were dissolved in 5% DMSO (vehicle) and injected intraperitoneally at the beginning of the experiment, and the same volume of DMSO was used in the vehicle and caerulein group. Mice were sacrificed 12 h after the first injection of caerulein.

### 2.4. Treatment with a Nrf2/HO-1 Pathway Inhibitor

A Nrf2 inhibitor (ML385) or a HO-1 inhibitor (ZnPP) was used to inhibit the Nrf2/HO-1 antioxidant pathway in vivo. ML385 was dissolved in 100% DMSO to prepare a stock solution and then diluted it into 5% DMSO solution with PBS before being used. ZnPP was dissolved as follows: 2.5 mg ZnPP was dissolved in 0.33 ml NaOH (0.2 M) in a dark room, and 0.2 M HCl was added to adjust the pH to 7.0. Finally, saline was added to 5 ml (0.5 mg/ml) [20, 21].

ML385 (30 mg/kg) or ZnPP (5 mg/kg) pretreatment was administered intraperitoneally 1 h before administration of caerulein, and the mice in the control group were treated with vehicle. In the MAP model, high-dose ISL (200 mg/kg) was administered after the first caerulein injection immediately to identify the underlying molecular mechanisms of ISL on AP.

### 2.5. Preparation of the SAP Model and ISL Intervention

A SAP mouse model was used in this study to further verify the protection of ISL on AP. The SAP model was induced by i.p injection of L-arginine (4 g/kg, 2 times, 1 h interval). After the first injection of L-arginine, 100 mg/kg ISL was injected intraperitoneally at 24 h, 36 h, 48 h, and 60 h, and mice were sacrificed at 72 h [22, 23].

### 2.6. Sample Collection and Preparation

All animals were anesthetized with i.p. administration of sodium pentobarbital (50 mg/kg) and sacrificed, and pancreatic and pulmonary tissues were dissected immediately. A portion of the tissues was fixed for histological analysis, and the remaining tissue was stored at −80°C for further investigation. Blood samples were obtained from the tail veins and stored at −80°C for analysis.

### 2.7. Oxidative Stress Injury Detection

Briefly, pancreatic tissues were homogenized in PBS and then centrifuged (12,000 rpm, 4°C, 30 min) to obtain supernatant. SOD, MDA, and GSH levels were determined according to the manufacturer's instructions [24, 25].

The content of ROS in pancreatic tissue was detected by DHE fluorescent probe. The fresh tissues of the pancreas were embedded in optimal cutting temperature (OCT) compound, and samples were cut into 7 *μ*m sections. An immunohistochemistry pen was used to draw a circle around the tissue for preventing the staining solution flow away. Tissues were incubated in the dark with DHE solution for 30 minutes at 37°C. Slides were placed in the PBS (pH = 7.4) and sloshed 3 times, each time for 5 min.

Then tissues were incubated by DAPI solution at room temperature for 10 min and sloshed again. Finally, the slides were observed under the fluorescence microscope.

### 2.8. Pancreatic Severity Assessment

Pancreatic and pulmonary tissues were collected and fixed in 4% paraformaldehyde and then embedded in paraffin blocks. Each paraffin block was stained with hematoxylin and eosin and used to assess the extent of tissue injury. The histopathological scoring analysis of the pancreas and lung was performed blindly by two pathologists according to previously described methods. The degree of histomorphologic damage of the pancreatic tissue was determined by the severity of edema, inflammatory cell infiltration, and acinar cell necrosis, and the degree of pulmonary tissue injury was scored by evaluating the severity of neutrophil infiltration, alveolar thickness, and alveolar congestion, as described in Supplementary Table 1-2. Blood samples were collected at different time points for serum enzymology (amylase and lipase) and proinflammatory cytokine (IL-1*β* and IL-6) detection. The analysis was performed according to the kit instructions.

### 2.9. Western Blot Detection

Pancreatic tissue lysates were prepared in saline-containing protease inhibitor and/or complete phosphatase inhibitor cocktail. The nucleoprotein was extracted using the reagents according to the instructions. The protein concentration was measured using a BCA kit and subjected to 10% SDS-polyacrylamide gel electrophoresis (PAGE), and the proteins were transferred to a PVDF membrane, blocked with 5% skim milk at room temperature for 2 h, and then incubated overnight at 4°C with primary antibodies against Nrf2 (1 : 1000 dilution), HO-1 (1 : 1000 dilution), Lamin B1 (1 : 1000 dilution), and *β*-actin (1 : 2000 dilution) in blocking buffer. Membranes were washed with TBST (3^∗^10 min) the next day and incubated with a secondary goat antimouse or goat antirabbit IgG horseradish peroxidase (HRP) antibody (1 : 10000 dilution) diluted in 5% (*w*/*v*) dry nonfat milk in TBST for 1 h at room temperature. Finally, membranes were washed with TBST (3^∗^10 min), developed using the ECL detection system (Santa Cruz Biotechnology), quickly dried, and exposed to ECL film. Image intensity was analyzed with ImageJ software.

### 2.10. Statistical Analysis

Statistical analysis was performed by SPSS 22.0 software. The results are presented as the mean ± standard deviation (SD). The biochemical measurements were analyzed with a one-way analysis of variance and Student-Newman-Keuls tests. For histological evaluation, the results were analyzed by a Mann–Whitney rank sum test, and *p* < 0.05 was considered statistically significant.

## 3. Results

### 3.1. Dynamic Changes of Oxidative Stress in the Pancreatic Tissues of Mice Induced by Caerulein

**Figure 1:**
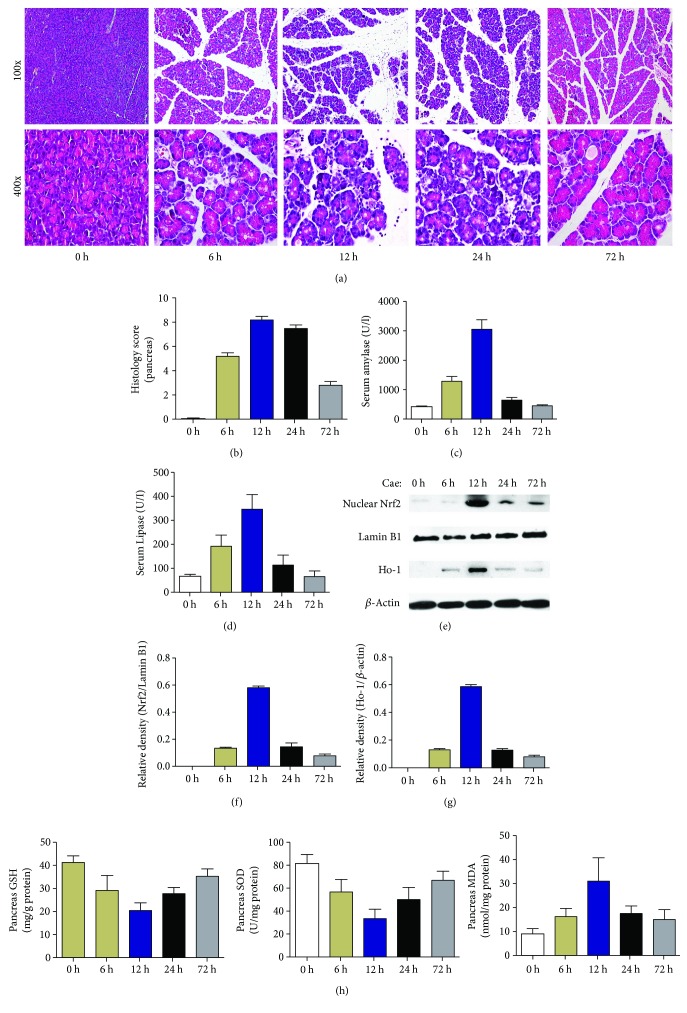
Dynamic changes of oxidative stress in the pancreatic tissues of mice induced by caerulein. (a) Representative HE staining of pancreas. (b) Histological scores of pancreas. (c-d) Serum levels of amylase and lipase. (e–g) Protein levels of nuclear Nrf2 and HO-1 in the pancreatic tissues were analyzed by Western blotting. Lamin B1 and *β*-actin were used as a control for protein loading. (h) Levels of oxidative stress products (SOD, MDA, and GSH) of pancreas. *n* = 6 each group.

Caerulein is a cholecystokinin mimic peptide that can induce MAP in rodents by stimulating the trypsin secretion of acinar cells. Given that the pathological characteristics and systemic inflammatory response mimic those of human AP, caerulein-induced MAP model is one of the most common murine models of AP [26, 27]. As shown in Figures 1(a) and 1(b), after administration of caerulein, the mouse pancreatic tissues presented with acute inflammatory injury, which manifested as edema, inflammatory cell infiltration, and acinar cell necrosis. Amylase and lipase serum levels are common biochemical markers of AP in mice, and the results showed that serum amylase and lipase increased gradually after AP onset, peaked at 12 h, and then dropped to the normal range at 72 h, as shown in Figures 1(c) and 1(d).

Additionally, the Nrf2 nucleoprotein and total HO-1 protein levels and the levels of oxidative stress products (SOD, MDA, and GSH) were detected to observe the dynamic changes of oxidative stress in pancreatic tissues of AP. The results showed that the Nrf2 nucleoprotein and total HO-1 protein were barely expressed in normal pancreatic tissues, while they increased significantly after AP onset and peaked at 12 h (Figures 1(e)–1(g)). Meanwhile, we found that MDA levels increased and SOD and GSH levels decreased after caerulein injection (Figure 1(h)). All the above oxidative stress changes were consistent with the pathological changes of pancreatic tissue and gradually decreased after 12 h. Consistent with previous studies, we found that oxidative stress was dynamically changed in pancreatic tissues of AP mice.

### 3.2. ISL Protected against the Histopathological Alterations of Pancreatic Tissue in Mice with MAP

**Figure 2:**
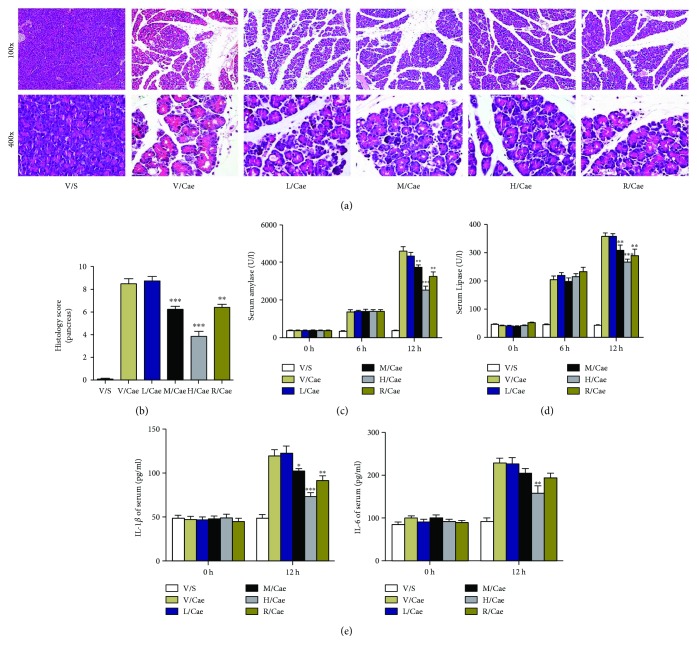
ISL attenuates caerulein-induced acute pancreatitis in mice. (a) Representative HE staining of pancreas. (b) Histological scores of pancreas. (c-d) Serum levels of amylase and lipase. (e) Changes in IL-1*β* and IL-6 levels in six groups. ^∗^ *p* < 0.05, ^∗∗^ *p* < 0.01, and ^∗∗∗^ *p* < 0.001 versus V/Cae group. *n* = 8 each group. V represents vehicle; S represents saline; L, M, and H represent low-dose (50 mg/kg), medium-dose (100 mg/kg), and high-dose ISL (200 mg/kg); R represents 100 mg/kg resveratrol.

Whether ISL has a protective effect on AP has not been previously reported. Based on the dynamic changes of oxidative stress in AP, mice were sacrificed 12 h after the first injection of caerulein. As shown in Figure 2(c), the serum amylase levels increased approximately 12.1-fold in the caerulein group at 12 h, but they decreased 7.7-fold in the high-dose ISL (200 mg/kg) administration group. Meanwhile, the lipase levels were increased approximately 3.9- and 2.8-fold in two groups. (Figure 2(d)). As a positive control drug, RES also significantly reduced the serum levels of amylase and lipase in MAP mice.

Then, we examined the degree of pancreatic tissue histopathological injury to assess the disease severity of MAP and found that both RES and ISL substantially alleviated the histological features of pancreatic tissue injury, resulting in decreased edema and inflammatory cell infiltration and alleviated acinar cell necrosis (Figures 2(a) and 2(b)). Additionally, the high-dose ISL (200 mg/kg) treatment was more effective than the medium-dose ISL (100 mg/kg) treatment, and we did not observe protective effects of ISL in the low-dose group (50 mg/kg) as shown in Figures 2(a) and 2(b). These results indicated that the protective effects of ISL on MAP were dose-dependent. The serum levels of proinflammatory cytokines such as IL-1*β* and IL-6 were hall markers of the systemic inflammatory responses of AP. Accordingly, the serum levels of IL-1*β* and IL-6 were detected by ELISA method. The results showed that the serum IL-1*β* and IL-6 were increased approximately 2-3-folds compared with the vehicle group and could be attenuated by RES and ISL administration, which was in accordance with the degree of pancreatic injury (Figure 2(e)).

### 3.3. ISL Reduced the Oxidative Stress Response of Pancreatic Tissue in Mice with MAP

**Figure 3:**
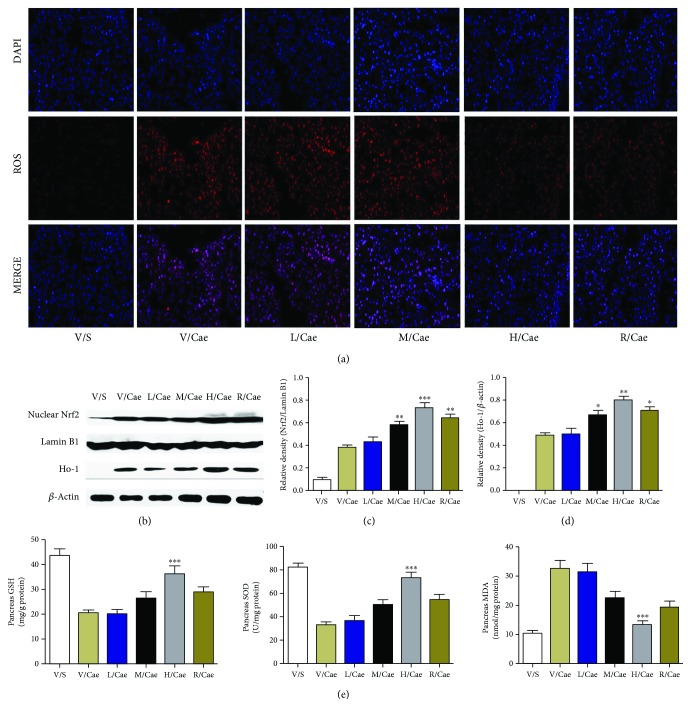
ISL reduced the oxidative stress response of pancreatic tissue in mice with MAP. (a) A representative immunofluorescence image of DHE. ROS activity was measured by fluorescent-labeled DHE staining. Stained sections of pancreas in magnification 200x. (b–d) Protein levels of nuclear Nrf2 and HO-1 in the pancreatic tissues were analyzed by Western blotting. Lamin B1 and *β*-actin were used as a control for protein loading. (e) Levels of oxidative stress products (SOD, MDA, and GSH) of pancreas. ^∗^ *p* < 0.05, ^∗∗^ *p* < 0.01, and ^∗∗∗^ *p* < 0.001 versus V/Cae group. *n* = 8 each group. V represents vehicle; S represents saline; L, M, and H represent low-dose (50 mg/kg), medium-dose (100 mg/kg), and high-dose ISL (200 mg/kg). R represents 100 mg/kg resveratrol.

After detecting the degree of pancreatic pathological injury and the changes in serum enzymology, we further observed the oxidative stress responses in pancreatic tissue after RES and ISL treatment. The DHE fluorescent probe was used to detect the content of ROS in pancreatic tissue [28]. ROS-specific staining was very low in the normal pancreatic tissue and accumulated significantly after pancreatic tissue injury induced by caerulein, whereas a markedly reduced signal was detected in both RES- and ISL- (100 mg/kg and 200 mg/kg) treated groups; moreover, the effect of ISL on ROS produce was dose-related (Figure 3(a)).

In addition, as shown in Figures 3(b)–3(d), the levels of Nrf2 nucleoprotein and total HO-1 protein were dynamically changed in pancreatic tissue 12 h after AP onset as determined by Western blot analyses. After RES and ISL administration, we observed that the expression levels of proteins in the Nrf2/HO-1 pathway substantially increased, which suggested that the Nrf2/HO-1 pathway was activated in RES and ISL-induced AP pathogenesis of mice. Additionally, the levels of MDA, SOD, and GSH in the pancreatic tissue of mice were detected, and as expected, the level of MDA was significantly decreased, and the levels of GSH and SOD were significantly increased after RES and ISL treatment (Figure 3(e)). The above results on the oxidative stress response were consistent with the pancreatic histopathological changes, indicating that ISL had an antioxidative stress effect and protected mice from AP through the oxidative stress pathway.

### 3.4. A Nrf2/HO-1 Pathway Inhibitor Counteracted the Protective Effect of ISL on Mice with MAP

**Figure 4:**
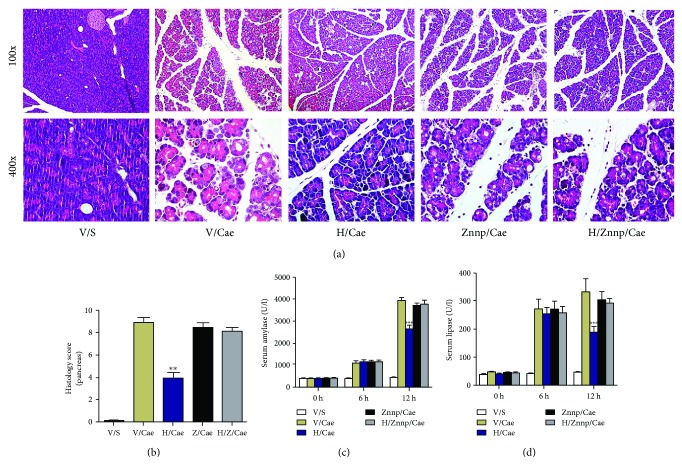
A HO-1 inhibitor, ZnPP, counteracted the protective effect of ISL on mice with MAP. (a) Representative HE staining of pancreas. (b) Histological scores of pancreas. (c-d) Serum levels of amylase and lipase. ^∗∗^ *p* < 0.01 and ^∗∗∗^ *p* < 0.001 versus V/Cae group. *n* = 12 each group. V represents vehicle; S represents saline; H represents high-dose ISL (200 mg/kg); Z represents HO-1 inhibitor, ZnPP.

To further identify the underlying mechanisms of ISL on AP in mice, we used a Nrf2 inhibitor (ML385) or HO-1 inhibitor (ZnPP) and high-dose ISL (200 mg/kg) for the following experiments. Based on previous reports, we used 30 mg/kg ML385 or 5 mg/kg ZnPP pretreatment and administered them intraperitoneally 1 h before caerulein treatment to inhibit the Nrf2 or HO-1 activity of mice, and mice in the control group were treated with vehicle [20, 21]. Similar to previous reports, ZnPP had no effect on AP pancreatic tissue injury of AP, while the protective effects of ISL on AP were blocked by pretreatment of ZnPP. The serum levels of enzymes (amylase and lipase) and the degree of injury in pancreatic tissue showed no significant differences between the ZnPP group and the ISL + ZnPP group (Figures 4(a)–4(d)).

**Figure 5:**
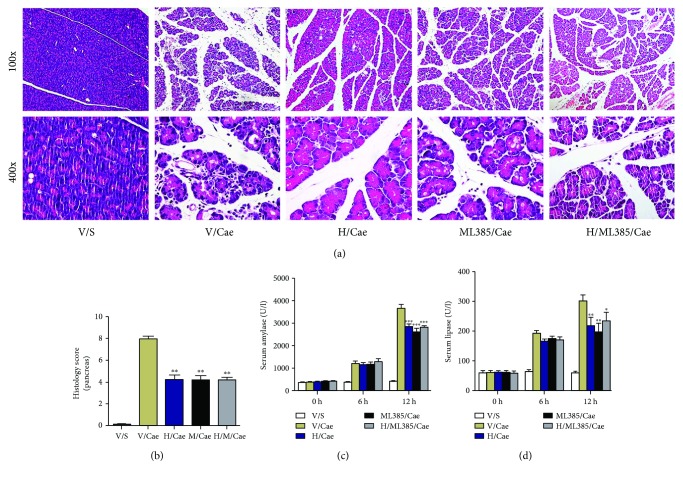
A Nrf2 inhibitor, ML385, counteracted the protective effect of ISL on mice with MAP. (a) Representative HE staining of pancreas. (b) Histological scores of pancreas. (c-d) serum levels of amylase and lipase. ^∗^ *p* < 0.05, ^∗∗^ *p* < 0.01, and ^∗∗∗^ *p* < 0.001 versus V/Cae group. *n* = 12 each group. V represents vehicle; S represents saline; H represents high-dose ISL (200 mg/kg); M represents Nrf2 inhibitor, ML385.

ML385, which has the same effect as ZnPP, was also administered to inhibit the activation of Nrf2 and blocked the protective effects of ISL on AP (Figures 5(a)–5(d)). However, to our surprise, ML385 could significantly alleviate the severity of AP in mice. Notably, using the Western blotting, we found that ML385 effectively inhibited the Nrf2 activity in pancreatic tissue of mice with MAP, and the effect of ISL on Nrf2 activity was offset by pretreatment of ML385, as shown in Supplementary Fig 1. Thus, the results confirmed that ISL could reduce the severity of AP by activating the Nrf2 pathway.

### 3.5. ISL Reduced the Severity of L-Arginine-Induced SAP in Mice

**Figure 6:**
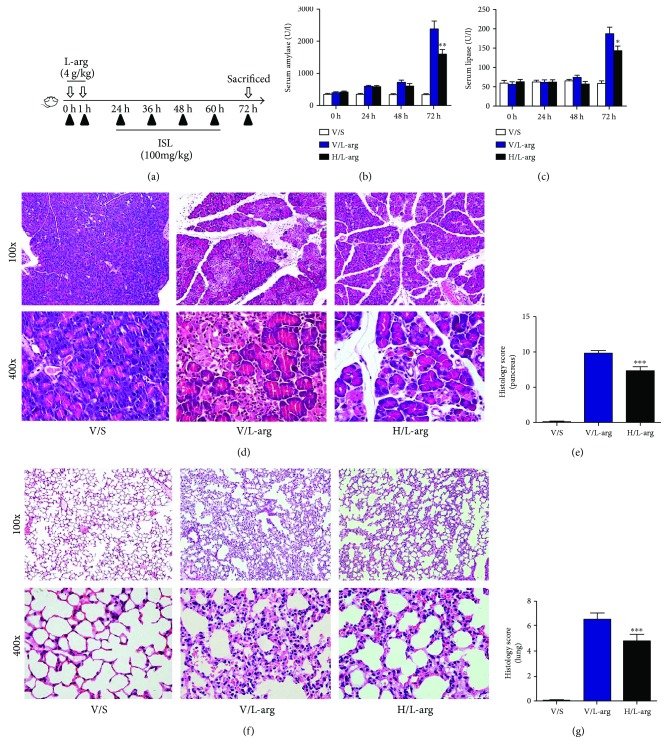
ISL reduced the severity of L-arginine-induced SAP in mice. (a) The experimental protocol with ISL in L-arginine-induced SAP model. (b-c) Serum levels of amylase and lipase. (d-e) Representative HE staining and histological scores of pancreas. (f-g) Representative HE staining and histological scores of the lung. ^∗^ *p* < 0.05, ^∗∗^ *p* < 0.01, and ^∗∗∗^ *p* < 0.001 versus V/L-arg group. *n* = 12 each group. V represents vehicle; S represents saline; H represents high-dose ISL (200 mg/kg).

To further confirm the protective effect of ISL on AP, we used a SAP mouse model induced by L-arginine. SAP was induced by injecting 4 g/kg/h L-arginine twice, and mice were treated with 100 mg/kg ISL intraperitoneally at 24 h, 36 h, 48 h, and 60 h after the first injection of L-arginine [29]. Unsurprisingly, the severity of pancreatic tissue injury, including acinar cell necrosis, edema, and inflammatory cell infiltration, was alleviated after ISL treatment (Figures 6(d) and 6(e)). The serum amylase and lipase levels were simultaneously detected and were shown to be consistent with the pathological results (Figures 6(b) and 6(c)). Considering that acute lung injury is the most common complication of SAP patients, we observed pathological changes in pulmonary tissues and found that pancreatitis-associated lung injury was reduced significantly after administration of ISL, as shown by the decreased alveolar edema and inflammatory cell infiltration and alleviated alveolar congestion (Figures 6(f) and 6(g)). All the above findings indicate that ISL could protect mice from SAP induced by L-arginine.

## 4. Discussion

By utilizing two different models of AP, we demonstrated for the first time that ISL has a protective effect on AP. AP is an acute noninflammatory disease of the digestive system, and the mortality rate of SAP remains high even with advances in current treatment approaches. There is still a lack of effective drugs for clinical therapy. Thus, identification of effective therapeutic drugs is urgently needed for improvement of AP treatment [30–32].

The present study focused on the protective effects of ISL on AP models in mice. ISL is a flavonoid component found in the hydrolysate of licorice root and has many potential medicinal properties. ISL inhibits tumor growth by promoting cell apoptosis. In addition, many studies found that ISL exhibits antioxidant, antiplatelet aggregation, and vasodilator effects [33–35]. Whether ISL can protect against AP is still unknown. In this study, we observed a significant protective effect of ISL on AP using two mouse models, in which the effect was primarily characterized by decreased serum amylase and lipase levels and pancreatic injury.

The pathogenesis of AP still remains unclear, while the oxidative stress pathway is recognized as one of the classical underlying mechanisms in AP. Clinical studies found that the significantly elevated plasma lipid peroxide levels and decreased SOD activity in SAP patients when compared to MAP patients. Moreover, the activity of serum antioxidant enzymes increased along with the recovery of AP. Ren et al. [36] found that the plasma level of MDA was an early predictor for the severity of AP, and other studies showed that antioxidant could reduce pancreatic necrosis. In animal models, it has been confirmed that pancreatic acinar cell injury could induce oxidative stress and release large amounts of hydrogen peroxide and superoxide substances, which caused ROS production and changes in MDA, GSH, and SOD levels. In this study, we found that in caerulein-induced acute pancreatic tissue injury, the levels of ROS and MDA remarkably increased as well as decreased GSH and SOD levels, which was consistent with previous studies [24, 25]. In addition, the associated protein levels in Nrf2/HO-1 pathway showed a mild elevation and we thought it was caused by the self-protective mechanism, which may not be enough to resist acute pancreatic injury. Antioxidative drugs, such as hemin, luteolin, and resveratrol, could further enhance the activation of Nrf2/Ho-1 pathway which has been proven to pose definitive anti-inflammatory effect [19, 21, 37]. It has been widely recognized that the generation of ROS and oxidative stress response occurs in the early phase of AP. Furthermore, the MDA level is positively correlated while the GSH and SOD levels are negatively correlated with degree of pancreatic tissue injury. ISL administration could inhibit the generation of ROS, promote the translocation of Nrf2, activate the Nrf2/Ho-1 pathway, and promote the expressions of protective oxidative products GSH and SOD while reducing MDA levels, which exerted a protective effect on AP via suppressing oxidative stress [38].

**Figure 7:**
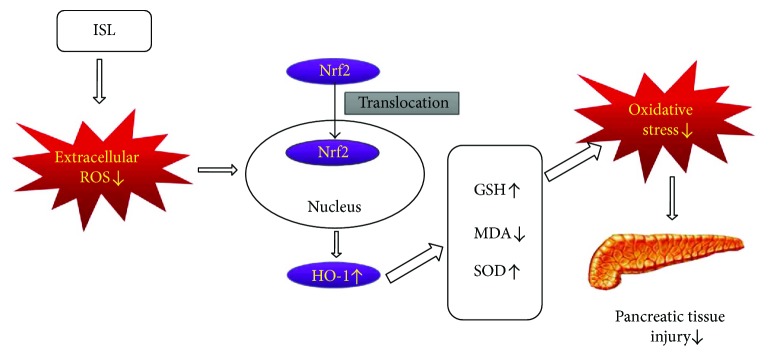
The protective mechanisms of ISL protect against pancreatic tissue injury in AP.

Nrf2/HO-1 pathway is the central regulator in cellular antioxidant responses. Nrf2 is an intranuclear antioxidant factor that interacts with the downstream HO-1 protein after entering the nucleus to activate the oxidative stress pathway [39, 40]. Activation of the Nrf2/HO-1 pathway has been reported to alleviate the severity of AP mice. In this study, we observed the dynamic changes of Nrf2/HO-1 expressions in caerulein-induced MAP model, and the activation of the Nrf2/HO-1 pathway was the most remarkable at 12 h after MAP induction. The results showed that ISL protected pancreatic injury in AP mice in a dose-dependent manner and was positively correlated with the degree of Nrf2/HO-1 activation, suggesting that ISL may protect AP through activation of Nrf2/HO-1, which was confirmed by two inhibitors of the Nrf2/HO-1 pathway (ML385 and Znpp). Znpp is an inhibitor of HO-1, and previous study has shown that Znpp could inhibit the activity of HO-1 in SAP model induced by caerulein combined with LPS [37]. ML385 is a novel and specific Nrf2 inhibitor discovered by the end of 2016, and it is reported that ML385 can inhibit Nrf2 transcriptional activity [20]. In addition to this, research on ML385 so far is rare and has not been reported in inflammatory diseases such as AP. In our study, ZnPP and ML385 were used as inhibitors of the Nrf2/HO-1 pathway to confirm the protective effect of ISL in AP. The results showed that intervention with ML385 or ZnPP could counteract the protective effect of ISL even when mice were given the high dose of ISL (200 mg/kg), which suggested that ISL protected against AP via activating Nrf2/HO-1 pathway in mice (Figure 7) [41, 42].

According to previous hypotheses, inhibition of Nrf2/HO-1 activity may aggravate the severity of AP or has no effect on AP in mice. Interestingly, our results showed that ML385 could alleviate the pancreatic injury. This result prompted us to confirm whether ML385 had an inhibitory effect on Nrf2 activation. Furthermore, we confirmed that ML385 actually inhibited nuclear translocation of Nrf2 in pancreatic tissue by using Western blot and speculated that the effect of ISL on Nrf2 was eliminated by pretreatment with ML385. While the mechanism for the protective effects of ML385 on AP remains unclear, ML385 is a novel small molecule compound and we know little about its biological activity until now. Hence, we surmised that ML385 may protect against AP through other nonproven pathways which have no effect on ISL. More research is needed to explore the biological activity of ML385 in inflammatory diseases in the future.

In conclusion, our findings demonstrated that ISL was effective in protecting against AP by inhibition of oxidative stress and modulation of the Nrf2/HO-1 pathway. These results suggest that ISL is a promising therapeutic treatment for AP in clinical practice.

## Supplementary Materials

Supplementary Materials Supplementary Table 1: histopathological scoring of pancreatic injury. Supplementary Table 2: histopathological scoring of lung injury. Supplementary Fig 1: a Nrf2 inhibitor, ML385, counteracted the protective effect of ISL on mice with MAP. (A-C) Protein levels of nuclear Nrf2 and HO-1 in the pancreatic tissues were analyzed by Western blotting. Lamin B1 and *β*-actin were used as a control for protein loading. *n* = 12 each group. V represents vehicle; S represents saline; H represents high-dose ISL (200 mg/kg); M represents Nrf2 inhibitor, ML385.
