# Supplementary material for: Isoliquiritigenin Ameliorates Acute Pancreatitis in Mice via Inhibition of Oxidative Stress and Modulation of the Nrf2/HO-1 Pathway
Source: Oxid Med Cell Longev. 2018 Apr 26;2018:7161592. doi: 10.1155/2018/7161592 (PMC5944199; doi:10.1155/2018/7161592)
Supplement: Supplementary Materials — Supplementary Table 1: histopathological scoring of pancreatic injury. Supplementary Table 2: histopathological scoring of lung injury. Supplementary Fig 1: a Nrf2 inhibitor, ML385, counteracted the protective effect of ISL on mice with MAP. (A-C) Protein levels of nuclear Nrf2 and HO-1 in the pancreatic tissues were analyzed by Western blotting. Lamin B1 and β-actin were used as a control for protein loading. n = 12 each group. V represents vehicle; S represents saline; H represents high-dose ISL (200 mg/kg); M represents Nrf2 inhibitor, ML385. [file 7161592.f1.pdf]

Supplementary Table 1. Histopathological Scoring of Pancreatic injury.

Score	Edema	Acinar necrosis	Inflammation
0	Absent	Absent	0-5 leukocytes/HPF
1	Diffuse expansion of interlobar septae	1-4 necrotic cells/HPF	6-15 leukocytes/HPF
2	Diffuse expansion of interlobubar septae	5-10 necrotic cells/HPF	16-25 leukocytes/HPF
3	Diffuse expansion of interacinar septae	11-16 necrotic cells/HPF (foci of confluent necrosis)	26-35 leukocytes/HPF
4	Diffuse expansion of intercellular spaces	>16 necrotic cells/HPF (extensive confluent necrosis)	>35 leukocytes/HPF or confluent microabscesses

Acinar cell necrosis and inflammatory infiltration were counted as the average number per 10 fields at magnification 400 ×.

Supplementary Table 2. Histopathological Scoring of Lung injury.

Score	Thickness of alveolar	Infiltration of neutrophils	Alveolar congestion
0	Absent	Absent	Absent
1	Discrete	Discrete	Small foci
2	Moderate	Moderate	Large foci
3	Severe	Severe	Diffuse

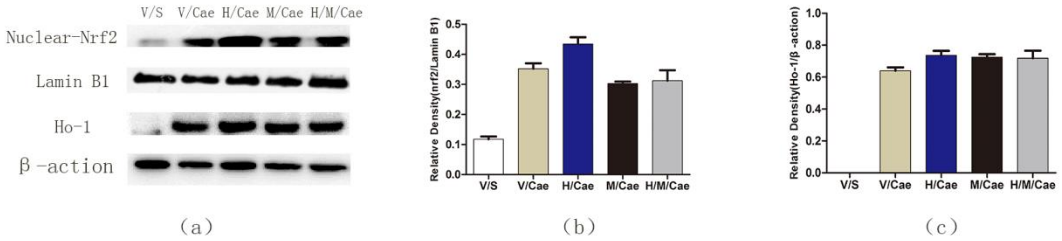

Supplementary figure 1. A Nrf2 inhibitor, ML385, counteracted the protective effect of ISL on mice with MAP. (a-c) Protein levels of Nuclear Nrf2 and HO-1 in the pancreatic tissues were analyzed by western blotting. Lamin B1 and β-Actin were used as a control for protein loading. n=12 each group. V represents vehicle; S represents saline; H represents high-dose ISL (200 mg/kg); M represents Nrf2 inhibitor, ML385.
